# Flavonoids as Natural Anti-Inflammatory Agents Targeting Nuclear Factor-Kappa B (NFκB) Signaling in Cardiovascular Diseases: A Mini Review

**DOI:** 10.3389/fphar.2019.01295

**Published:** 2019-10-31

**Authors:** Ker Woon Choy, Dharmani Murugan, Xin-Fang Leong, Razif Abas, Aspalilah Alias, Mohd Rais Mustafa

**Affiliations:** ^1^Department of Anatomy, Faculty of Medicine, Bioscience and Nursing, MAHSA University, Jenjarom, Malaysia; ^2^Department of Pharmacology, Faculty of Medicine, University of Malaya, Kuala Lumpur, Malaysia; ^3^Centre for Craniofacial Diagnostics and Biosciences, Faculty of Dentistry, Universiti Kebangsaan Malaysia, Kuala Lumpur, Malaysia; ^4^Department of Human Anatomy, Faculty of Medicine and Health Sciences, Universiti Putra Malaysia, Serdang, Malaysia; ^5^Department of Basic Sciences and Oral Biology, Faculty of Dentistry, Universiti Sains Islam Malaysia, Kuala Lumpur, Malaysia; ^6^Centre for Natural Product research and Drug Discovery (CENAR), Wellness Research Cluster, University of Malaya, Kuala Lumpur, Malaysia

**Keywords:** flavonoids, nuclear factor-kappa B signalling, anti-inflammatory, cardiovascular diseases, natural compounds

## Abstract

Cardiovascular diseases (CVDs) such as angina, hypertension, myocardial ischemia, and heart failure are the leading causes of morbidity and mortality worldwide. One of the major transcription factors widely associated with CVDs is nuclear factor-kappa B (NFκB). NFκB activation initiates the canonical and non-conical pathways that promotes activation of transcription factors leading to inflammation, such as leukocyte adhesion molecules, cytokines, and chemokines. Flavonoids are bioactive polyphenolic compounds found abundantly in various fruits, vegetables, beverages (tea, coffee), nuts, and cereal products with cardiovascular protective properties. Flavonoids can be classified into six subgroups based on their chemical structures: flavanones, flavones, flavonols, flavan-3-ols, isoflavones, and anthocyanidins. As NFκB inhibitors, these flavonoids may modulate the expression of pro-inflammatory genes leading to the attenuation of the inflammatory responses underlying various cardiovascular pathology. This review presents an update on the anti-inflammatory actions of flavonoids *via* inhibition of NFκB mechanism supporting the therapeutic potential of these natural compounds in various CVDs.

## Introduction

Cardiovascular diseases (CVDs) represent the major burden of mortality and morbidity in the developed countries (Benjamin et al., 2017). The most common pathogeneses of CVDs are inflammatory processes (Ruparelia et al., 2017). Various transcription factors are related to inflammatory responses in CVDs such as T-bet (Haybar et al., 2019), signal transducer and activator of transcription 3 (STAT3) (Kurdi et al., 2018), interferon regulatory factors (IRFs), activator protein 1 (AP-1) (Smale and Natoli, 2014), and transcription factor Bcl11b (Daher et al., 2019). However, the key player in the regulation of inflammation is the transcription factor nuclear factor kappa B (NFκB) (Van Der Heiden et al., 2010). The inhibition of NFκB pathway has been demonstrated to show beneficial effect in various CVDs including hypertension (Koeners et al., 2016), myocardial infraction (Zhao et al., 2017), and arteriosclerosis (Wang et al., 2016). These findings support that targeted inhibition of NFκB appears to be a promising strategy in reducing cardiovascular complications.

Flavonoids are plant polyphenolic compound derivatives from natural origin found in fruits, grains, vegetables, roots, bark, flowers, stems, tea, and wine (Zeinali et al., 2017). Non-plant natural products such as mushrooms and honey, plant extracts, plant juices, plant powders, and essential oils have shown to possess anti-inflammatory activities and many of these plant natural products have polyphenols as their major compound (Khalil and Sulaiman, 2010; Azab et al., 2016). However, the protective effects of flavonoid in CVDs *via* inhibition of NFκB are yet to be reviewed. Therefore, in this mini-review, we focused on the anti-inflammatory actions of flavonoids *via* inhibition of NFκB mechanism in CVDs.

## Flavonoids and Its Subclass

Flavonoids are categorized into six subclasses depending on its chemical structures: flavones, flavonols, flavanones, isoflavones, flavan-3-ols, and anthocyanidins (Panche et al., 2016).

Flavones are found abundant in flowers, fruits, and leaves such as red peppers, celery, parsley, chamomile, mint, and ginkgo biloba (Manach et al., 2004). The most studied flavones are luteolin, apigenin, and tangeritin (Manach et al., 2004).

Flavonols such as kaempferol, myricetin, quercetin, rutin, fisetin, silymarin, and isorhamnetin are ubiquitous in foods such as saffron, onions, kale, lettuce, tomatoes, apples, grapes, berries, red wine, and tea (Pollastri and Tattini, 2011).

Flavanones widely present in all citrus fruits, which gives the bitter taste of the juice and its peel. Oranges, lemons, and grapes are rich sources of flavanones and major compounds are hesperitin, naringenin, and eriodictyol (Barreca et al., 2017).

Isoflavones are unique in that they resemble estrogen in structure and, therefore, are classified as phytoestrogens. There are found abundantly in soy products such as tofu, roasted soy nuts, and miso (Marzocchella et al., 2011).

Flavan-3-ols, also called as dihydroflavonols, include catechin, epicatechin, gallocatechin, epigallocatechin, epicatechingallate, epigallocatechingallate, and procyanidin (Alkhalidy et al., 2018). The most commonly associated food with the flavan-3-ol compounds is black and green tea and fruits such as bananas, apples, blueberries, peaches, and pears (Osakabe, 2013).

Anthocyanins are rich in outer cell layers of fruits such as merlot grapes, raspberries, cranberries, red grapes, strawberries, blueberries, bilberries, and blackberries. The most commonly studied anthocyanins are cyanidin, delphinidin, malvidin, pelargonidin, and peonidin (Khoo et al., 2017).

## Nfκb Induced Inflammation and CVDs

**Figure 1:**
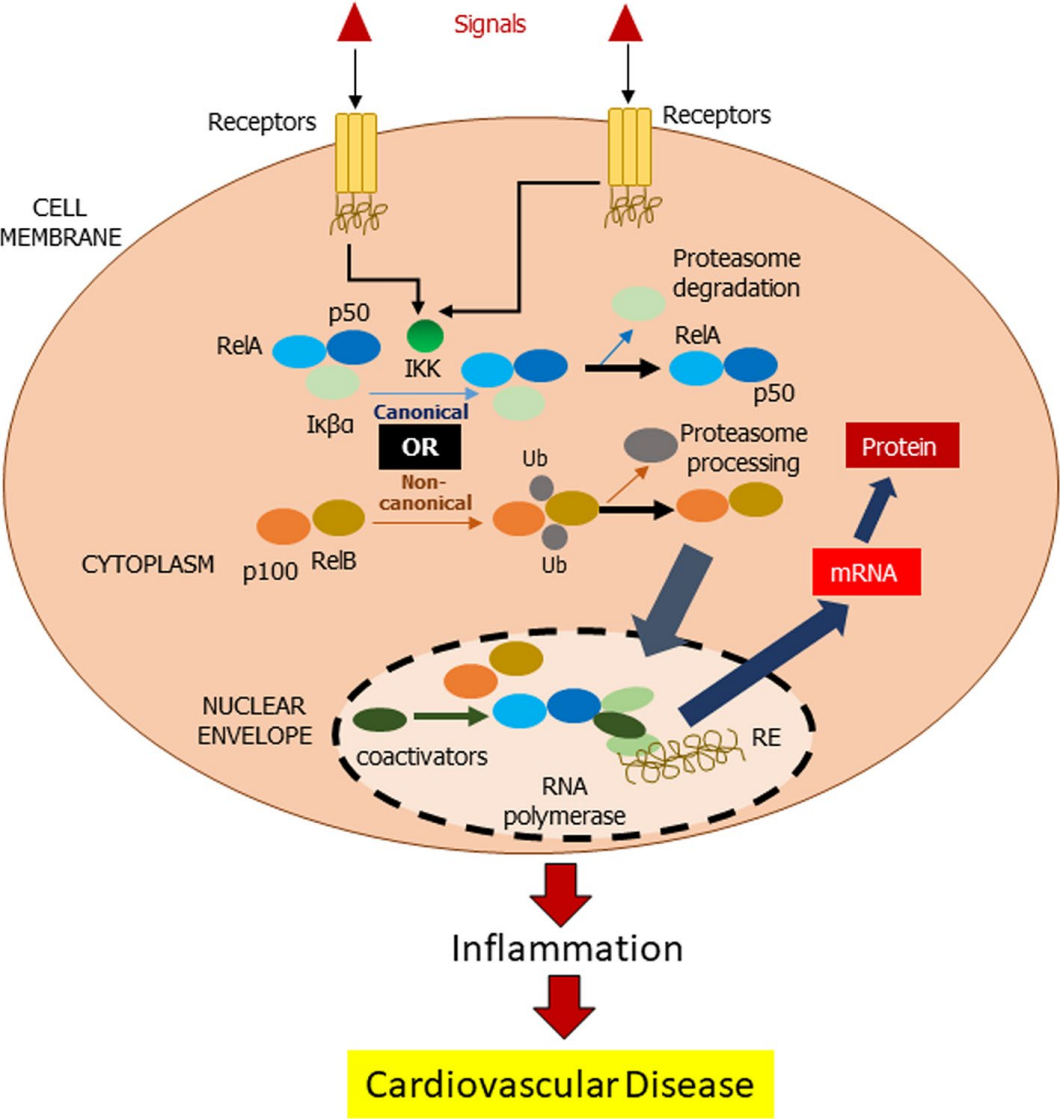
Mechanism of NFκB action. In inactivated state, NF-κβ, which consists of Rel and p50 proteins, is located in the cytosol complexed with the inhibitory protein Iκβα. IκB kinase (IKK) is activated by extracellular signals *via* membrane receptors. Subsequently, IKK phosphorylates the Iκβα protein resulting in ubiquitination of Iκβα and eventually by the proteasome for Iκβα degradation (canonical pathway). In non-canonical pathway, RelB favors the activation of NF-κβ *via* RelB. Activated NF-κβ is further translocated into the nucleus for DNA bindings, called response elements (RE). The DNA/NF-κβ complex attracts coactivators and RNA polymerase, which transcribe the DNA into mRNA resulting in a cell transformation.

There are a few cellular redox pathways involved in the development of the chronic inflammatory CVD, which includes NFκB. NFκB is a transcription factor that activates inhibitor of kappa B (IκB) kinase in the cytosol upon being stimulated by inflammatory stimuli (Brasier, 2010). Subsequent signaling pathways *via* canonical or non-canonical lead to migration of NFκB toward the nucleus and hence initiates the targeting gene such as pro-inflammatory cells, monocytes, macrophages, and T and B cells (**Figure 1**).

The canonical NFκB pathway responds rapidly to stimuli and activates NFκB, which increases pro-inflammatory cytokines such as interleukin (IL)-1β, IL-6, and tumor necrosis factor alpha (TNF-α), which results in cell apoptosis. TNF-α receptor signaling plays an important role in the canonical pathway of NFκB in cell death *via* Jun N-terminal kinases (JNK), p38, and caspase 8 cascades (Ghosh et al., 2009). Furthermore, NFκB also activates angiotensin II, endothelin-1, and phenylephrine as hypertrophic agonist *via* IκB degradation and p65 nuclear translocation.

A central signaling component of the non-canonical NFκB pathway is NFκB-inducing kinase, which induces p100 phosphorylation through kinase IKKα in a slow manner (Sun, 2017). Ligands of a subset of tumor necrosis factor receptor (TNFR) superfamily members are typical inducers of the non-canonical NFκB pathway (Shih et al., 2011).

## Anti-Inflammatory Effects of Flavonoids in CVDs *via* Modulation of NFκB Signaling

### Quercetin

Quercetin or 3, 3′, 4′, 5, 7-pentahydroxyflvanone that falls into the category of flavonol is widely found in plants such as Ginkgo biloba, *Hypericum perforatum*, and *Sambucus canadensis* as well as vegetables such as apples, berries, grapes, onions, shallots, and tomatoes (Li et al., 2016).

In a clinical study involving patients with chronic systemic inflammation (CSI) in stable coronary artery disease (CAD), quercetin showed anti-inflammatory effects with reduction in indicators of CSI (Chekalina et al., 2018). Quercetin decreased IL-1β and TNF-α levels in blood serum, in addition to decreasing the transcriptional activity of NFкB in blood mononuclear cells (Chekalina et al., 2018). In leptin-induced inflammation model using human umbilical vein endothelial cells (HUVECs), quercetin significantly suppressed the upregulation of Ob-Ra (leptin receptor) expression, ERK1/2 phosphorylation, NFкB, and TNF-α (Indra et al., 2013). Furthermore, in a neonatal rat cardiac fibroblast, quercetin inhibited TNF-α, IL-1β, and IL-6 secretion by inhibiting the activation of NFкB and Akt induced by lipopolysaccharide (LPS) (Tang et al., 2014).

### Luteolin

Luteolin or 3’, 4’, 5, 7-tetrahydroxyflavone is one of the most prevalent flavones widely found in fruits and vegetables such as carrots, cabbages, parsley, broccoli, celery, and apple skins (Weng and Yen, 2012).

In an *in-vivo* sodium fluoride-induced hypertensive model, administration of luteolin increased nitric oxide (NO) bioavailability, reversed prolongation of QT and QTc intervals, and reduced the expressions of kidney injury marker 1 (Kim-1), NFκB, and cardiac troponin I (CTnI), which eventually normalized the blood pressure (Oyagbemi et al., 2018a). Previous study in neonatal rat cardiac myocytes exposed to LPS showed luteolin reduced the TNF-α levels in the medium, downregulated the TNF-α mRNA in myocytes, inhibited degradation of IκB-β and nuclear translocation of NFκB, as well as reduced NFκB DNA binding, proposing the therapeutic potential of luteolin the management of inflammation-related myocardial diseases (Lv et al., 2011).

### Fisetin

Fisetin or 3, 3c,4c,7-tetrahydroxyflavone is a bioactive molecule found in fruits such as strawberry, apple, persimmon, and grape and vegetables such as onion and cucumber (Arai et al., 2000).

Garg et al. (2019) reported the protective effect of fisetin against isoproterol-induced myocardial injury by suppressing myocardial injury markers, creatine kinase-muscle/brain (CK-MB), lactate dehydrogenase (LDH), and inflammatory markers (TNF-α and IL-6) in the blood serum as well as normalization of histological and ultrastructure of the heart. In addition, fisetin regulated the balance between pro- or anti-oxidants and pro- or anti-apoptotics proteins in the myocardial tissue (Garg et al., 2019). These protective effects of fisetin are attributed to the downregulation of receptor for advanced glycation end products (RAGE) and NFκB (Garg et al., 2019).

Fisetin attenuated the development of diabetic cardiomyopathy by attenuating the expression of myocardial NFκB and the pro-inflammatory cytokines IL-1β, IL-6, and TNF-α in the heart of diabetic rats. These result in reduction of cardiac function markers such as CK-MB, LDH, and cTn as well as normalization heart morphology (Althunibat et al., 2019).

### Apigenin

Apigenin, a flavone, is found widely available in fruits and vegetables, such as grapefruits, oranges, celeries, and onions (Ren et al., 2018).

In LPS-treated macrophages, apigenin has been shown to reduce toll-like receptor 4 (TLR-4), MyD88, and p-IκB-α expression levels *via* nuclear NFκB p65 signaling pathway (Ren et al., 2018). Similarly, in LPS-challenged apoE-/- mice, treatment with apigenin increased expression of ATP binding cassette A1 (ABCA1), which alleviated extra lipid accumulation, reduced miR-33, TLR-4, and NFκB p65 levels, lessened the macrophages and smooth muscle cell contents in the atherosclerotic region, and improved plasma lipid profile (Ren et al., 2018). These results suggested that apigenin attenuates atherogenesis by inhibition of nuclear NFκB p65 that up-regulates ABCA1-mediated cholesterol efflux (Ren et al., 2018).

Apigenin was also shown to improve cardiac dysfunction and fibrosis in diabetic cardiomyopathy. Apigenin blunted the activity of NFκB and downregulated the activity of caspase3 accompanying with decreasing oxidative stress marker, glutathione peroxidase (GSH-Px), malondialdehyde (MAD), and superoxide dismutase (SOD) (Huangjun et al., 2016).

### Isoliquiritigenin

Isoliquiritigenin is extracted from root of licorice and has been used traditionally for the treatment of inflammatory or pulmonary diseases (Peng et al., 2015). In HUVECS exposed to TNF-α, isoliquiritigenin blocked the involvement of NFкB at the transcriptional levels, and thus attenuated the downstream expression of VCAM-1, E-selectin, THP-1 monocyte adhesion, IкB-α, and PECAM-1, suggesting the protective effects of isoliquiritigenin through NFкB-dependent mechanisms (Kwon et al., 2007). In angiotensin II induced hypertension model, isoliquiritigenin attenuated inflammation cytokines including IL-1β and TNF-α, excessive deposition of extracellular matrix, and oxidative stress-induced apoptosis *via* nuclear factor E2-related factor 2 (Nrf2) and NFκB pathways (Xiong et al., 2018).

### Rutin

Rutin is a flavonol that presents in buckwheat and citrus fruits. In a sodium fluoride-induced hypertensive rats, administration of rutin reduced blood pressure elevation by enhancing NO bioavailability *via* down-regulation of NFκB expression and up-regulation of Nrf2 (Oyagbemi et al., 2018b).

In carfilzomib-induced cardiotoxicity in rat, rutin protected against myocardial hypertrophy by upregulating IκB-α and downregulating NFκB expression, resulting in attenuation of β-myosin heavy chain, reduction in B-type natriuretic peptide mRNA expressions, and normalization of cardiac muscle ﬁber morphology (Imam et al., 2017).

In addition, rutin increased activities of Nrf, decreased activation of NFκB in human embryonic kidney reporter cell line, and preserved relaxation of fetal placental arteries derived from human chorionic plate (Sthijns et al., 2017).

In high mobility group box 1 (HMGBI)-induced inflammatory response in HUVECs, rutin attenuated NFκB and ERK1/2, which, in turn, reduced IL-6 and TNF-α levels (Yoo et al., 2014). Up-regulation of VCAM-1, intercellular adhesion molecule-1 (ICAM-1), and E-selectin induced by HMGB1 were similarly inhibited by rutin, suggesting that the protective effect of rutin on vascular inflammation is by inhibiting the HMGB1 and NFκB pathways.

In LPS-induced inﬂammation in HUVECs, rutin reversed barrier disruption, expression of cell adhesion molecules, and adhesion and migration of monocytes in endothelial cells. The barrier protective effects of rutin were linked to a down-regulation of TNF-α, deactivation of NFκB, and reduced phosphorylation of IκB-α (Lee et al., 2012).

### Chrysin

Chrysin (5,7-dihydroxyﬂavone) is a flavone, which is found in the blue passion ﬂower, honey, and propolis (Mantawy et al., 2017). Chrysin prevented doxorubicin (DOX)-induced cardiomyopathy including disturbance of cardiac conduction, increased serum cardiac markers and histopathological alteration in heart of rat *via* downregulation of NFκB, mitogen-activated protein kinase (MAPK), suppression of AKT pathway and its upstream activator, vascular endothelial growth factor (VEGF) (Mantawy et al., 2017).

In a rat model of monocrotaline-induced pulmonary arterial hypertension (PAH), chrysin reduced right ventricular systolic pressure and mean pulmonary artery pressure. In addition to suppression of right ventricular remodeling, chrysin abolished increased expression of collagen I, collagen III, and NFκB (Li et al., 2015).

In isoprenaline-induced myocardial injury in rats, chrysin relieved hemodynamic and ventricular dysfunction as well as reduced ultrastructural myocardial damage *via* inhibition of NFκB, IκKβ expression, and TNF-α level as well as increased peroxisome proliferator-activated receptor-gamma (PPAR-γ) expression (Rani et al., 2016).

In a rat model of myocardial infarction, fibrosis in the interstitial and perivascular regions and expression of collagen was reduced following chrysin treatment (Yang et al., 2018). This effect is associated with increased PPAR-γ expression and decreased NFκB expression *via* inhibition of IκKβ phosphorylation, leading to reduction of matrix metalloproteinase-2 (MMP-2), MMP-9 levels, and suppression of activator protein 1 (AP-1) level.

### Genistein

Genistein under the subgroup of an isoflavone [4′,5,7-trihydroxyisoflavone,5,7-dihydroxy-3-(4-hydroxyphenyl)-4-H-1-benzopyran-4-one] is primarily found in soy-based foods, legumes, and red clover. In homocysteine-induced endothelial cell inﬂammatory injury, genistein prevented endothelial damage *via* blockade of activation of NFκB, expression of inflammatory cytokine and adhesion molecule, IL-6, and ICAM-1 (Han et al., 2015).

Xu et al. (2019) explored the effect of genistein on angiotensin II-induced vascular smooth muscle cell inflammation. Angiotensin II-induced expression of NFκB, C-reactive protein (CRP), MMP-9, phosphorylation of ERK1/2 and p-38, which lead to atherosclerotic inflammatory damage, were reversed following genistein treatment. Furthermore, genistein enhanced expression of PPAR-γ, suggesting cardiovascular protective effect by the isoflavone is through regulation of p38/ERK1/2-PPARγ-NFκB signaling pathway (Xu et al., 2019).

### Silymarin

Silymarin is a flavonolignan extracted from the milk thistle. Silymarin augmented relaxation of pulmonary arteries isolated from a lung ischemia-reperfusion (I/R) injury model (Jin et al., 2016). Vascular protective effect of silymarin is due to inhibition of NFκB, thus suppressing the serum concentration of inflammatory cytokines and reducing protein expression of hypoxia inducible factor-1α (HIF-1α) and iNOS.

Silibinin, a major active constituent of silymarin, was able to reduce the abnormal size of cardiac myocytes and prevent hypertrophy by alleviating the production of epidermal growth factor receptor (EGFR) (Ai et al., 2010). Silibilin exerted its anti-inflammatory effect by suppressing the activation of NFκB stimulated by angiotensin II in cardiac myocytes or in the aortic banding male mice. Furthermore, silibilin interfered with the phosphorylation and degradation of IκB-α and activation of IκKβ *in vivo*.

### Kaempferol

Kaempferol (3,4′,5,7-tetrahydroxyflavone) is a flavonol that is present widely in fruits, vegetables, and herbs, including grapes, tomatoes, and tea. In cardiac fibroblasts stimulated with LPS, kaempferol decreased release of pro-inflammatory cytokines by inhibiting AKT phosphorylation and NFκB activation (Tang et al., 2015). In isoprenaline-induced cardiac damage, kaempferol improved the hemodynamic and left ventricular functions in male rats, which abated the increased serum concentration of CK-MB and LDH, preserved the morphology of myocardium, and reduced the levels of pro-inflammatory cytokines (Suchal et al., 2016a). Similarly, kaempferol prevented cardiac damage by inhibiting the protein expression of NFκB, p38, and JNK (Suchal et al., 2016b) suggesting that cardioprotective and anti-inflammatory action of kaempferol was associated with NFκB signaling pathway.

**Table 1** summarizes the effects and mechanisms of action of flavonoids in CVD.

**Table 1:** Effect of flavonoids in CVDs.

No	Flavonoids	Models	Mechanisms	Reference
1	Quercetin	Clinical study: CAD patients	↓ NFкB, IL-1β, TNF-α, IkBα	(Chekalina et al., 2018)
		*In vitro*: leptin-induced inflammation and endothelial dysfunction in HUVECs	↓ ERK1/2 phosphorylation, NFкB, TNF-α	(Indra et al., 2013)
		*In vitro*: neonatal rat cardiac fibroblast inflammatory	↓ NFкB, TNF-α, IL-1β, IL-6, AKT	(Tang et al., 2014)
2	Luteolin	*In vivo*: NaF-induced hypertension	↑NO ↓ Nrf2, Kim-1, NFκB, CTnI	(Oyagbemi et al., 2018a)
		*In vitro*: Neonatal rat cardiac myocytes inflammatory	↓ NFκB, TNF-α, ↑ IκB-β	(Lv et al., 2011)
3	Fisetin	*In vivo*: Isoprenaline-induced cardiac ischemic injury	↓NFκB, RAGE, TNF-α, IL-6, CK-MB, LDH	(Garg et al., 2019)
		*In vivo*: Hyperglycemia-induced cardiac injury	↓NFκB, IL-1β, IL-6, TNF-α	(Althunibat et al., 2019)
4	Apigenin	*In vitro*: LPS-treated macrophages *In vivo*: LPS-challenged apoE-/- mice	↓ NFκB p65, TLR-4, MyD88, p-IκB-α ↑ ABCA1	(Ren et al., 2018)
		*In vivo*: diabetic cardiomyopathy	↓ NFκB, caspase3, GSH-Px, MDA, SOD	(Huangjun et al., 2016)
5	Isoliquiriti-genin	*In vitro*: TNF-α induced inflammation in HUVECs	↓ NFкB, VCAM-1, E-selectin, THP-1 monocyte adhesion, IкB-α, PECAM-1	(Kwon et al., 2007)
		*In vivo*: Angiotensin II-induced hypertension	↓ NFкB, IL-1β and TNF-α Nrf2,	(Xiong et al., 2018)
6	Rutin	*In vivo:* Sodium fluoride induced hypertension in rat	↓ NFκB ↑ Nrf2	(Oyagbemi et al., 2018b)
		*In vivo:* carfilzomib-induced cardiotoxicity in rat	↓ NFκB ↑ IκB-α	(Imam et al., 2017)
		*In vitro*: hydrogen peroxide induced oxidative stress in HUVECS	↓ NFκB ↑ Nrf2	(Sthijns et al., 2017)
		*In vitro*: HMGBI-induced inflammatory in HUVECS	↓ NFκB, ERK1/2, TNFα, IL-6, ICAM-1, VCAM-1, E-selectin	(Yoo et al., 2014)
		*In vitro*: LPS induced inflammation in HUVECS	↓ NFκB, IκB-α, TNF-α, ICAM-1, VCAM-1, E-selectin	(Lee et al., 2012)
7	Chrysin	*In vivo:* DOX-induced cardiotoxicity in rat	↓ p38, JNK, NFκB ↑ VEGF, AKT	(Mantawy et al., 2017)
		*In vivo:* monocrotaline-induced pulmonary arterial hypertension in rat	↓ NFκB	(Li et al., 2015)
		*In vivo:* ISO-induced myocardial injury in rat	↑ PPAR-γ ↓ NFκBp65, IκK-β, TNF-α	(Rani et al., 2016)
		*In vivo*: MI in rat	↑ PPAR-γ ↓ NFκB, IκK-β, MMP-2, MMP-9, AP-1	(Yang et al., 2018)
8	Genistein	*In vitro*: Homocysteine-induced endothelial cell inﬂammation in HUVECS	↓ NFκBp65, IL-6, ICAM-1	(Han et al., 2015)
		*In vitro*: angiotensin II-induced VSMCs inflammation	↑ PPAR-γ ↓ ERK1/2, p38, NFκB, CRP, MMP-9	(Xu et al., 2019)
9	Silymarin	*In vivo:* I/R injury in rat	↓ NFκB, HIF-1α, iNOS, TNFα, IL-1β, IL-6	(Jin et al., 2016)
		*In vivo:* Cardiac hypertrophy model in mouse	↓ NFκB, EGFR, IκB-α, IκKβ	(Ai et al., 2010)
10	Kaempferol	*In vitro*: LPS+ATP stimulated cardiac fibroblasts inflammation	↓ AKT, NFκBp65, TNF-α, IL-1β, IL-6, IL-18	(Tang et al., 2015)
		*In vivo*: I/R cardiac injury in rat	↓ p38, JNK, NFκBp65, TNF-α, IL-6	(Suchal et al., 2016a; Suchal et al., 2016b)

## Conclusion

The actions of flavonoids in mitigating inflammation by modulation of NFкB offer potential agents for the treatment of CVDs. However, several of these actions reported *in vitro* may yet to be fully recognized due to their low bioavailabilities following oral administration (Hollman and Katan, 1998; Thilakarathna and Rupasinghe, 2013). Flavonoids have shown promising results in reducing atherosclerosis in several animal experimental models; however, conflicting results were reported in human clinical trials (Arts and Hollman, 2005; Zordoky et al., 2015). The low bioavailability and clinical efficacy of flavonoids are attributed to their poor absorption, metabolism by the metabolizing enzymes in the intestine and liver, and structural modifications by the colonic bacteria remain as the major problems. Continuous investigation is required to enhance the bioavailability and efficacy of the flavonoids to tap the full potential of these natural agents.

## Author Contributions

All authors contributed to the writing. KC, DM, and MM conceived, designed, and revised the manuscript.

## Funding

This study was funded by Government Agency grant GA001-2017, MAHSA University and USIM research project code: PPPI/FPG/0118/051000/15618 Universiti Kebangsaan Malaysia and Universiti Putra Malaysia. The funding agencies played no role in the design of the study and collection, analysis, and interpretation of data and in writing the manuscript, which are fully the responsibilities of the authors.

## Conflict of Interest

The authors declare that the research was conducted in the absence of any commercial or financial relationships that could be construed as a potential conflict of interest.

The reviewer ZJ declared a shared affiliation, with no collaboration, with one of the authors, XFL, to the handling editor at time of review
